# Structural Characterization of Lignin in Four Cacti Wood: Implications of Lignification in the Growth Form and Succulence

**DOI:** 10.3389/fpls.2018.01518

**Published:** 2018-10-17

**Authors:** Jorge Reyes-Rivera, Marcos Soto-Hernández, Gonzalo Canché-Escamilla, Teresa Terrazas

**Affiliations:** ^1^Programa de Botánica, Colegio de Postgraduados en Ciencias Agrícolas, Texcoco, Estado de México, Mexico; ^2^Centro de Investigación Científica de Yucatán, Unidad de Materiales, Mérida, Mexico; ^3^Departamento de Botánica, Instituto de Biología, Universidad Nacional Autónoma de México, Mexico City, Mexico

**Keywords:** Cactaceae, wood lignin structure, S-rich lignin, dimorphic wood, structural protection, succulence, evolutionary-adaptive processes

## Abstract

Wood lignin composition strongly depends on anatomical features and it has been used as a marker for characterizing major plant groups. Wood heterogeneity in Cactaceae is involved in evolutionary and adaptive processes within this group; moreover, it is highly correlated to the species growth form. Here we studied the lignin structure from different types of woods in four Cactaceae species with different stem morphologies (*Pereskia lychnidiflora*, tree/fibrous wood; *Opuntia streptacantha* and *Pilosocereus chrysacanthus*, tree/succulent fibrous wood; *Ferocactus hamatacanthus*, cylindrical stem/dimorphic wood) in order to determine their relationship with the wood anatomy in an evolutionary-adaptive context. Dioxane lignin was isolated and analyzed by pyrolysis coupled with gas chromatography and mass spectrometry (Py-GC/MS), two-dimensional nuclear magnetic resonance spectroscopy (2D-NMR) and attenuated total reflectance-Fourier transform infrared spectroscopy (ATR-FTIR). The main linkages are the β-O−4′ ether (67–85%), the β-β′ resinol (10–26%) and the β-5′ and α-O−4′ linkages of the phenylcoumaran structures (≤7%). Spirodienone structures have a considerable abundance (5%) in the dimorphic wood of *F. hamatacanthus*. In addition, low contents (≤3%) of α,β-diaryl ether, α-oxidized β-O−4′ ether and dibenzodioxocin structures were found. The sinapyl- and coniferyl acetates are not part of the wood lignin in any of the studied species. The low (≤5%) γ-acetylation in the *F. hamatacanthus* and *P. chrysacanthus* wood lignin is here interpreted as an evidence of a high specialization of the wood elements in the conduction/storage of water. The lignin of the studied Cactaceae is composed predominantly of guaiacyl and syringyl units (S/G: 0.9–16.4). High abundance of syringyl units (62–94%) in three of the four species is considered as a defense mechanism against oxidative agents, it is a very conspicuous trait in the most succulent species with dimorphic wood. Furthermore, it is also associated with ferulates and the herein called γ-acetylated guaiacyl-syringaresinol complexes acting as nucleation sites for lignification and as cross-links between lignin and carbohydrates at the wide-band tracheid-fiber junctions.

## Introduction

Lignin is a phenolic biopolymer derived from hydroxycinnamyl alcohols that differ in their degree of methoxylation: p-coumaryl, coniferyl and sinapyl alcohols, although other monolignols have been recently proposed (Lu and Ralph, 2002; del Río et al., 2007; Ralph, 2010; Chen et al., 2013; Carlos Del Río et al., 2017). It is formed under simple chemical control by bimolecular radical coupling reactions and its structure is highly dependent on the nature of monolignols and the cellular characteristics of the lignified tissue (Ralph J. et al., 2004; Bonawitz and Chapple, 2010; Umezawa, 2010; Vanholme et al., 2010). Lignin imparts structural strength to plants to keep the stem upright, also confers resistance to the cellular wall to withstand the negative pressures generated during transpiration in the conducting elements and plays an important role in the defense against pathogens (Bonawitz and Chapple, 2010; Weng et al., 2010; Barros et al., 2015; Meents et al., 2018). Thus, it has been regarded that the acquisition of lignin biosynthesis represents a fundamental adaptation which gave plants the ability to colonize terrestrial ecosystems and that its evolution has been parallel to that of tracheophytes (Xu et al., 2009; Bonawitz and Chapple, 2010; Lucas et al., 2013; Tohge et al., 2013). On the other hand, the composition of wood lignin has been used as a marker that characterizes major groups within tracheophytes (Baucher et al., 1998; Lupoi et al., 2015). In gymnosperms, where wood consists exclusively by tracheids and axial parenchyma, lignin is mainly composed by guaiacyl units (G) with small amounts of p-hydroxyphenyl units (H), derived from the coniferyl and p-coumaryl alcohols, respectively (Bonawitz and Chapple, 2010). In angiosperms, where the diversity of cell types in wood is higher, lignin is composed by moieties of syringyl (S) units, derived from sinapyl alcohol, and G units (Bonawitz and Chapple, 2010; Barros et al., 2015), with only small amounts of H units.

**Figure 1:**
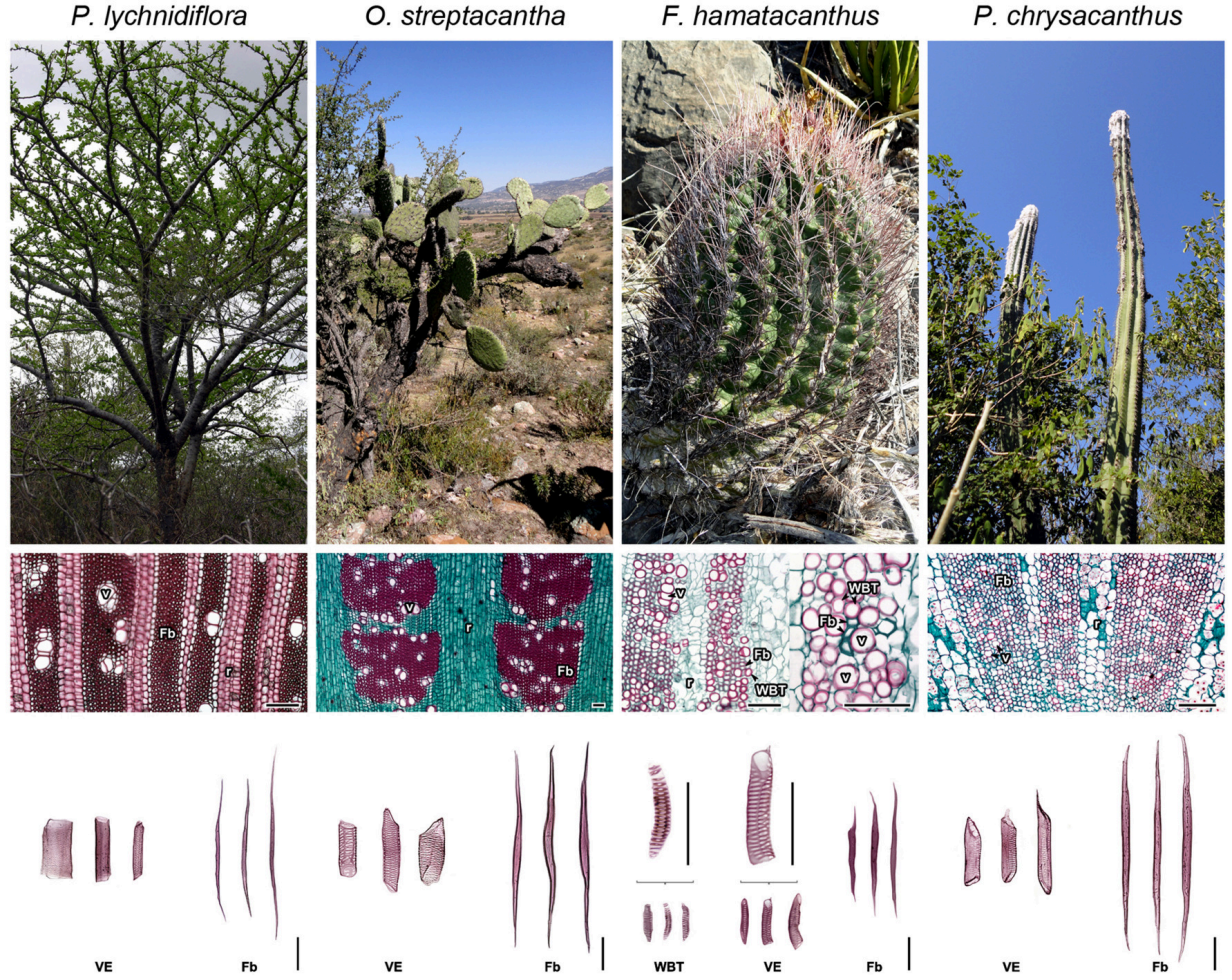
Morpho-anatomical features of the four species of Cactaceae studied. Columns ordered from left to right based on the evolutionary trends of the subfamilies within Cactaceae. Superior row: stem morphology in adults. Middle row: wood anatomy, transversal sections. Inferior row: wood macerations, obtained as described in Reyes-Rivera et al. (2017). Fb, fibers; r, rays; v, vessels; VE, vessel elements; WBT, wide-band tracheids. Bars = 150 μm.

In an evolutionary context, the Cactaceae family is considered by several authors as one of the most surprising radiations of succulent plants in the New World angiosperms, due to its physiological, anatomical and metabolic characteristics (Nobel and Hartsock, 1986; Ogburn and Edwards, 2009; Ocampo and Columbus, 2010; Arakaki et al., 2011). It has been hypothesized that the evolution of succulent growth form was greatly influenced by internal anatomical novelties in the stem, particularly those of the wood (Altesor et al., 1994; Carlquist, 2001; Mauseth, 2006). Such features involve an increased capacity of wood for water retention (Mauseth, 2006), very specialized tracheary elements with a limited secondary cell wall extension (Figure 1; Carlquist, 2001; Terrazas and Mauseth, 2002; Grego-Valencia et al., 2015; Reyes-Rivera et al., 2017) and a decreased lignification rate, which translates into a low wood accumulation (Altesor et al., 1994; Reyes-Rivera et al., 2017). Some of these characteristics are more or less conspicuous depending on the phylogeny: in Opuntioideae and Cactoideae, two of the most recently derived subfamilies with a predominance of succulent forms, diverse wood configurations have been reported (cambial variants *sensu*; Carlquist, 2001). These varies from monomorphic woods, with predominance of just one cell type, to polymorphic woods where similar proportions of more than one cell type, or alternatively, several changes in the predominant cell types occur (Mauseth and Plemons, 1995; Terrazas and Arias, 2002; Mauseth, 2006; Vázquez-Sánchez and Terrazas, 2011; Reyes-Rivera et al., 2017). In addition, there are species where the fibers, typical wood elements in woody dicots, are scarce or absent, instead occur an abundance of a very specialized cell type with limited extension of the secondary cell wall, called wide-band tracheid (WBT, Figure 1). On the other hand, in Pereskioideae, the most ancestral subfamily with non-succulent stems, wood is completely fibrous, and in some species such as *Pereskia lychnidiflora*, WBTs are never present (Terrazas and Mauseth, 2002; Mauseth, 2004; Reyes-Rivera et al., 2017). An intriguing aspect is that lignin of many dimorphic woods is distinguished by being exceptionally rich in S units (97% in *F. hamatacanthus*, as determined by nitrobenzene oxidations) and by having a heterogeneous composition (Reyes-Rivera et al., 2015). In contrast, the fibrous species show more homogeneous composition patterns (Reyes-Rivera et al., 2015) and lack KNOX transcription factors that are present in dimorphic species, factors related to dimorphic wood change (Reyes-Rivera et al., 2017). Thus, the different types of wood in Cactaceae are an excellent system to study the relationship between lignification and some evolutionary aspect of the growth form and other adaptive aspects concerning dimorphic wood structure.

In this work, we analyse lignin structure of different types of wood in four species of Cactaceae, in order to understand its relationship to the wood anatomical features and stem morphology in an evolutionary-adaptive context. Lignin with low structural modification was isolated following procedures previously described (Evtuguin et al., 2001; Rencoret et al., 2015) and analyzed by 2D-NMR, Py-GC/MS, and ATR-FTIR. The results make evident three important aspects: (1) confirm different alternatives in Cactaceae wood for the enhancement to the water conduction/retention; (2) there is a great correlation between wood lignin structure, wood anatomical features and stem morphology; (3) the relationship between lignification and defense against oxidative damage is here considered.

## Materials and methods

### Species selection

Due to the conservation status of some of the species and based on the consistency observed in previous studies (Reyes-Rivera et al., 2015, 2017), this work was focused to four representative species of the three main Cactaceae subfamilies. The selection was made based on the high correspondence between wood anatomy and growth forms all over Cactaceae. The species and their morpho-anatomical characteristics are shown in Figure 1. *Pereskia lychnidiflora* A.P. de Candolle (Pereskioideae; subfamily with fibrous wood), is the most ancestral tree representative of all the selected species, with non-succulent stems and WBTs completely absent; *Opuntia streptacantha* Lemaire (Opuntioideae) is a representative of the transition toward the succulent forms where WBTs may or may not be present and the succulence of the wood begins to be noticeable; *Ferocactus hamatacanthus* (Muehlenpf.) Britton & Rose (Cactoideae; tribe Cacteae, with predominantly succulent forms, small in size, with mainly dimorphic wood or with predominance of WBTs) representative with dimorphic wood and lignin exceptionally rich in S units; *Pilosocereus chrysacanthus* (F. A. C. Weber ex K. Schum.) Byles & G. D. Rowley (Cactoideae; tribe Cereeae), belongs to one of the most recently derived groups within Cactaceae, with fibrous wood and stems of great height, moderately succulent. Adult and healthy individuals were used to obtain wood from the base of the stem, the closest to the vascular cambium as possible, since mature wood is found there (Reyes-Rivera et al., 2015; Reyes-Rivera and Terrazas, 2017).

### Preparation of plant material

Fresh wood samples were first air-dried and later dried in a convection oven at 50°C for 48 h. Due to the anatomical characteristics of wood in these species (Figure 1), milled wood was used as reported by Evtuguin et al. (2001) in order to minimize changes in lignin structure, since excessive milling can modify it considerably (Fujimoto et al., 2005). Reduction of the particle size was performed using a Willey mini-mill, model 3383-L10 (Thomas Scientific, Swedesboro, NJ, United States), the samples were sieved and the portion retained between the 40–60 meshes was used (422 μm pore size). Due to the succulence of the wood, it retains diverse compounds; therefore, the extractives-free wood (E-FW) was obtained by exhaustive sequential Soxhlet extraction, with benzene-ethanol (2:1), ethanol (96%), and distilled water (Reyes-Rivera et al., 2015; Reyes-Rivera and Terrazas, 2017).

### Isolation of dioxane lignin

Dioxane lignin (DL), considered as representative of native lignin due to its relatively low structural modification (Rencoret et al., 2015), was extracted here from the E-FW following procedures previously described (Evtuguin et al., 2001; Rencoret et al., 2015), with some modifications. 4 g of E-FW were placed inside a three-mouth flask with 40 mL of a dioxane/water solution (9:1, v/v) acidulated with HCl, equivalent to 0.2 M. The material was refluxed at 90°C for 40 min, under a nitrogen atmosphere. Then, the mixture was allowed to cool in a nitrogen atmosphere to around 50°C; the liquid phase was carefully decanted and kept separately. The solid residue was extracted twice more, in the same manner, with 30 mL of the acidulated solution of dioxane/water for 30 min. A fourth extraction of the solid residue was performed using an acid-free dioxane/water solution (9:1, v/v). The liquid phases of the four extractions were concentrated separately at 40°C under reduced pressure, around 2 mL. The concentrates were combined and lignin was precipitated with 260 mL of cold distilled water. The lignin was collected by centrifugation, extracted with diethyl ether, washed twice with distilled water and it was finally freeze-dried.

### Py-GC/MS analysis

Pyrolysis of DL (100 μg, in duplicate) was performed using a pyrolyzer model PY-3030S (Frontier Laboratories, Japan) coupled to a GC/MS system, model QP2010 (Shimadzu Scientific, Japan), equipped with an UA+ −5 column (5% diphenyldimethyl polysiloxane; 60 m × 0.25 mm, film thickness 0.25 mm; Frontier Laboratories, Japan). The pyrolysis was performed at 500°C. The GC oven temperature was programed from 50°C (1 min), to 100°C (at 30°C/min) and finally to 300°C (at 10°C/min). The final temperature was held for 10 min. The GC/MS interface was kept at 300°C and He was used as carrier gas. The interpretation of the mass spectra was made based on previous studies reported in the literature (Faix et al., 1990; Ralph and Hatfield, 1991; del Río et al., 2004) and by comparison with the NIST libraries. The syringyl/guaiacyl ratio (S/G) was calculated based on the integrated area of the chromatograms, corresponding to the sum of the derivatives of G and S units. The presence of γ-acylated units of the lignin was determined following the procedure described by del Río et al. (2004), using samples of E-FW. Approximately 100 μg of powdered E-FW were pyrolyzed. The pyrolysis was performed at 610°C for 4 s. The chromatograph was programmed from 40°C (1 min) to 300°C at a rate of 6°C/min. The final temperature was kept for 20 min. The injector temperature was kept at 280°C while the GC/MS interface was kept at 300°C.

### 2D-NMR analysis

The 2D-RMN analysis were made at 25°C in a Bruker Avance III HD 400 MHz spectrometer (Bruker, Karlsruhe, Germany), equipped with a BBI 400 MHz probe, with a Z gradient. Approximately 40 mg of DL were dissolved in 0.75 mL of DMSO-d6 according to the method previously described by Rencoret et al. (2015). The 2D-NMR spectra were registered using the Bruker's pulse program “hsqcetgp” with phase-sensitive ge-2D HSQC echo-antiecho. The cosine squared bell apodization was applied for both ^1^H and ^13^C dimensions. HSQC spectra were processed with MestReNova, v11.0.4-18998 software (Mestrelab Research, Spain). Prior to Fourier transformation, the data matrices were zero-filled to 1024 points in the ^13^C dimension. For correct peak integration, the spectra were previously baseline-corrected with the default option. The central peak of the deuterated solvent was used as an internal reference (DMSO, δ_C_/δ_H_ 39.52/2.49). 2D-NMR HSQC cross-peaks were assigned by comparison with those reported in the literature (Ralph S. A. et al., 2004; Rencoret et al., 2008, 2015; Ralph and Landucci, 2010; del Río et al., 2012; Wen et al., 2013; Constant et al., 2016). A semi-quantitative analysis was made with the HSQC spectra intensities as described by Constant et al. (2016), the integration was made separately for the different spectra regions. The relative abundances of the different inter-unit linkages were estimated in the aliphatic oxygenated region (δ_C_/δ_H_ 50-90/2.7-5.6). The C_α_-H_α_ cross-peaks were always used, except for the α-oxidized β-O−4′ structures (Aox) and cinnamyl alcohol end-groups (I), for which the C_β_-H_β_ and C_γ_-H_γ_ cross-peaks were used, respectively. The relative abundances of the aromatic units were estimated in the aromatic/unsaturated region (δ_C_/δ_H_ 100-160/6.0-8.0). For correct estimation of the S/G ratio, the C–H pairs in similar environments were used (Mansfield et al., 2012). The entire value of the C_2_-H_2_ cross-peaks in the G units and half of the value from the C_2,6_-H_2,6_ cross-peaks in the S units were used to calculate their molar percentages (G + S = 100). For the cinnamyl aldehyde end-groups (J) and stilbenes (St), half of the value from the C_2,6_-H_2,6_ and C_α, β_-H_α, β_ cross-peaks were used, respectively.

### ATR-FTIR analysis

ATR-FTIR spectra were obtained from the DL samples using an Agilent Cary 630 FTIR spectrometer (Agilent Technologies, Santa Clara, CA, United States) equipped with an ATR diamond unit. 100 μg of DL, milled in an agate mortar, were placed directly in the ATR unit. Each spectrum was obtained using MicroLab PC software (Agilent Technologies, Santa Clara, CA, United States), at a spectral range of 650–4,000 cm^−1^, through 32 scans (15 s per reading), with a resolution of 4 cm^−1^ and the Happ-Genzel apodization. A total of five experiments were averaged for each species. The spectra were processed using Origin Pro 2016, vb9.3.226 software (OriginLab Corporation, Northampton, MA, United States). Each spectrum was baseline-corrected, tracing the baseline between the valleys at 894, 1,171, 1,536, and 1,762 cm^−1^. The deconvolution of the spectra was made by peak adjustment using the default Gaussian function. For the chemometric analysis, only the region between 750 and 1,750 cm^−1^ was used. In order to calculate the S/G ratio, the integrated areas corresponding to the vibrations of the S and G aromatic rings were used, peaks around 1,324 and 1,270 cm^−1^, respectively.

### Chemometric analysis

With the data from the Py-GC/MS, 2D-NMR (HSQC) and ATR-FTIR experiments, combined matrices were made to compare the four species regarding each variable (i.e., height of species, wood type, growth form, molar and relative abundances for each structure or derivative, height of the peaks and S/G ratio values). The SPSS v.18.0 software (SPSS Inc., Chicago, IL, United States) was used to perform the descriptive statistical analysis, principal component analysis, hierarchical cluster and Pearson correlation analyses. In the principal component analysis, two types of matrices were made: those including the discrete variables and others excluding them. Interpretation of the results was made comparing both analyses.

## Results

### Py-GC/MS analysis

**Figure 2:**
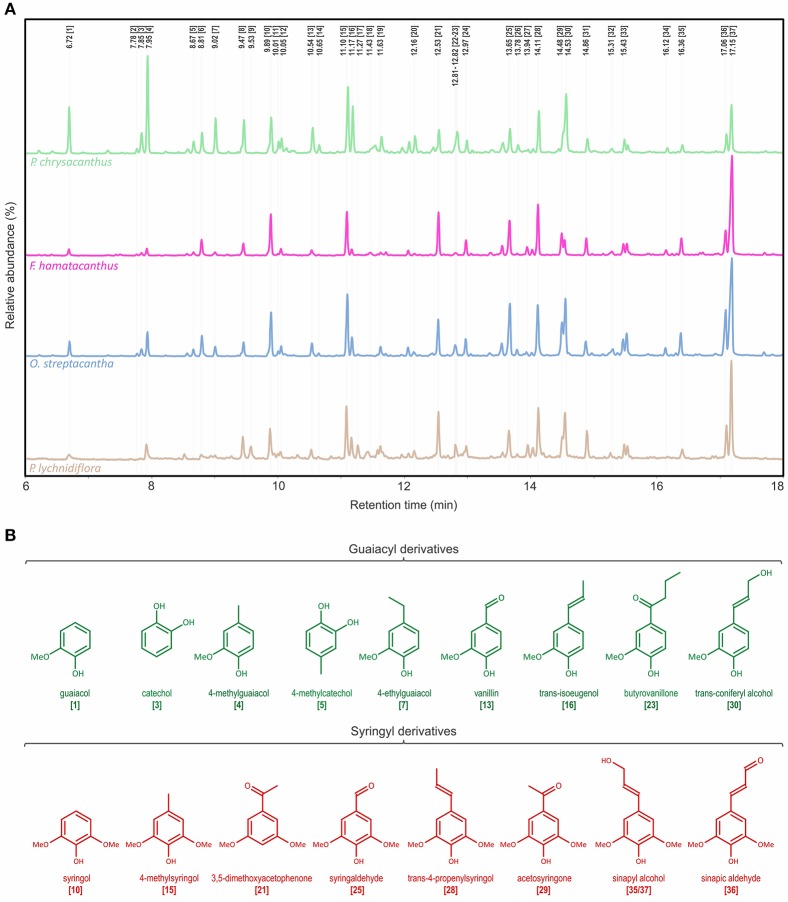
Characterization of the wood lignin by Py-GC/MS for the species of Cactaceae studied. **(A)** Py-GC/MS chromatograms for the DL of the mature wood. **(B)** Structures of the main phenolic derivatives. Identities and relative abundances of the released compounds are listed in Table 1.

**Figure 3:**
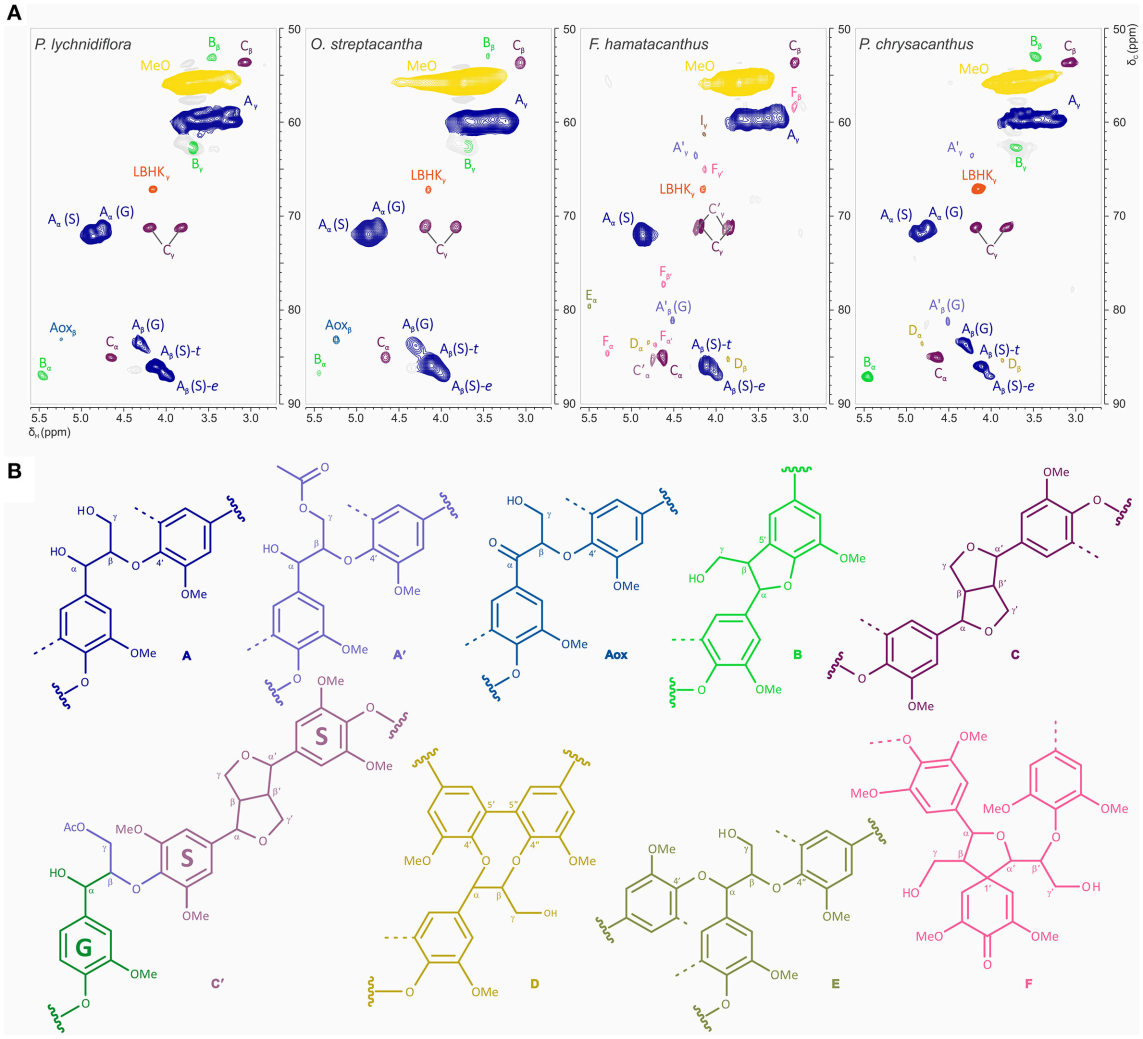
Structural features of the aliphatic oxygenated region in the wood lignin for the species of Cactaceae studied. **(A)** Side-chain region (δC/δH 50–90/2.7–5.6 ppm) in the HSQC spectra. **(B)** Structures of the principal inter-unit linkages identified. Assignments for the cross-peaks in each structure are shown in Table 2.

The Py-GC/MS analysis allowed knowing the predominant units in the DL. Py-GC/MS chromatograms of DL and the structures of the predominant derivatives are shown in Figure 2. Identities, origin and relative abundances of the released compounds by Py-GC/MS of the DL, as well as the S/G ratio, are given in Table 1. Lignin of the Cactaceae species studied here consists predominantly of G and S units. No derivatives of H units were found. The most abundant compounds were those derived from S units, such as 4-methylsyringol (compound 15, Table 1; Figure 2), 3,5-dimethoxyacetophenone [21], *trans*-4-propenylsyringol [28], sinapic aldehyde [36] and *trans*-sinapyl alcohol [37]. In addition, derivatives from the guaiacyl units were observed, such as guaiacol [1], 4-methylguaiacol [4], *trans*-isoeugenol [16] and *trans*-coniferyl alcohol [30]. Based on the S/G ratio calculated, it was observed that in three of the four studied species there was a predominance of S units (Table 1). In *F. hamatacanthus, O. streptacantha* and *P. lychnidiflora* a high percentage of *trans*-sinapyl alcohol (11–26%) was obtained. Whereas, in *P. chrysacanthus*, with a higher G lignin content (61%), the main derivative was 4-methylguaiacol (12%).

**Table 1:** Identities and molar abundances of the main compounds released by Py-GC/MS of the DL.

**Peak**	**RT (min)^a^**	**CAS**	**Compound**	**MW**	**Origin**	***P. lychnidiflora***	***O. streptacantha***	***F. hamatacanthus***	***P. chrysacanthus***
1	6.72	90-05-1	Guaiacol	124.14	G	1.55	2.02	0.67	5.46
2	7.78	18102-31-3	2-Methoxy-3-methylphenol	138.16	G	0.00	0.44	0.00	0.65
3	7.85	120-80-9	Catechol	110.11	G	0.00	1.01	0.68	2.48
4	7.95	93-51-6	4-Methylguaiacol	138.16	G	2.61	3.24	0.75	11.32
5	8.67	452-86-8	4-Methylcatechol	124.14	G	0.00	0.98	0.77	1.49
6	8.81	934-00-9	3-Methoxycatechol	140.14	G	1.53	2.77	1.50	2.52
7	9.02	2785-89-9	4-Ethylguaiacol	152.19	G	1.47	1.33	0.41	4.18
8	9.47	7786-61-0	4-Vinylguaiacol	150.17	G/FA	3.40	1.89	1.27	3.97
9	9.53	621-59-0	Isovanillin	152.15	G	2.40	0.00	0.00	0.00
10	9.89	91-10-1	Syringol	154.16	S	4.25	5.78	7.45	4.26
11	10.01	1941-12-4	3-Allylguaiacol	164.20	G	1.63	0.74	0.73	1.51
12	10.05	2033-89-8	3,4-Dimethoxyphenol	154.16	C-Cc ^b^	1.84	0.00	0.00	1.87
13	10.54	121-33-5	Vanillin	152.15	G	2.08	1.77	0.62	3.09
14	10.65	5912-86-7	*cis*-Isoeugenol	164.20	G	1.38	0.47	0.00	1.09
15	11.10	6638-05-7	4-Methylsyringol	168.19	S	6.56	8.14	7.82	7.75
16	11.17	5932-68-3	*trans*-Isoeugenol	164.20	G	3.30	2.54	0.73	5.55
17	11.27	2785-87-7	4-Propylguaiacol	166.22	G	2.52	0.33	0.00	0.00
18	11.43	498-07-7	Levoglucosan	162.14	Carb.	1.86	0.00	0.97	0.35
19	11.63	498-02-2	Acetovanillone	166.17	G	2.42	1.32	0.42	2.06
20	12.16	2380-78-1	Homovanillyl alcohol	168.19	G	1.95	0.66	0.00	2.22
21	12.53	39151-19-4	3,5-Dimethoxyacetophenone	180.20	S	5.99	4.86	8.00	2.85
22	12.81	100377-63-7	Vanillic acid hydrazide	182.18	G	2.40	0.00	0.35	0.00
23	12.81 / 12.82	64142-23-0	Butyrovanillone	194.23	G	0.00	1.55	0.00	2.69
24	12.97	6627-88-9	Methoxyeugenol	194.23	S	2.44	2.33	2.94	1.62
25	13.65	134-96-3	Syringaldehyde	182.17	S	4.07	6.92	6.89	2.97
26	13.78	0-00-0	4-((1e)-3-Hydroxy-1-propenyl)-2-methoxyphenol	180.00	G	1.59	0.94	0.00	1.10
27	13.94	4497-40-9	Methylconiferylaldehyde	192.21	G	2.55	0.67	0.95	0.57
28	14.11	20675-95-0	*trans*-4-Propenylsyringol	194.23	S	6.39	6.74	8.97	5.02
29	14.48	2478-38-8	Acetosyringone	196.20	S	5.86	4.49	4.27	0.65
30	14.53	32811-40-8	*trans*-Coniferyl alcohol	180.20	G	1.47	7.57	1.68	6.95
31	14.86	4385-56-2	Homosyringic acid	212.20	S	4.04	2.00	3.31	1.78
32	15.31	530-57-4	Syringic acid	198.17	S	1.74	1.01	0.98	0.67
33	15.43	19037-58-2	Syringylpropanone	210.23	S	2.60	2.29	2.29	1.72
34	16.12	63543-12-4	5-(3-Hydroxypropyl)-2,3-dimethoxyphenol	212.24	UD	0.00	1.18	1.12	0.76
35	16.36	537-33-7	*cis*-Sinapyl alcohol	210.23	S	2.10	3.09	3.56	1.10
36	17.06	87345-53-7	Sinapic aldehyde	208.21	S	4.58	6.10	5.03	2.35
37	17.15	20675-96-1	*trans*-Sinapyl alcohol	210.23	S	11.27	12.83	25.83	5.71
					% G	37	33	11	61
					% S	63	67	89	39
					S/G ^c^	1.7	2.1	8.5	0.7

*Carb., Carbohydrates; FA, ferulates; G, Guaiacyl units; MW, molecular weight; RT, Retention time; S, Syringyl units; UD, undefined*.

a *The number for each compound and its respective retention time correspond to the peaks indicated in Figure 1*.

b *Compounds formed by cleavage of the C-C linkages*.

c *The sum of all derivatives of the S and G units was used to calculate the S/G ratio, except for F. hamatacanthus where 4-vinylguaiacol was not taken into account since it can be derived from the ferulates. Compounds of undefined origin were omitted*.

#### Determination of γ-acylation by Py-GC/MS

The Py-GC/MS chromatograms of the E-FW are shown in Figure S1. Identities, origin and relative abundances of the released compounds, as well as the S/G ratio, are given in Table S1. In the region of lignin derivatives, the same compounds obtained in the pyrolysis of the DL were observed (Table 1 and Table S1; Figure 2, Figure S1). Coniferyl acetate and sinapyl acetate were not found among the derivatives of the pyrolysis. The S/G ratio obtained for each species was highly consistent with that obtained for the DL by Py-GC/MS (Table 1 and Table S1), except for *F. hamatacanthus* where the S/G ratio was underestimated.

### 2D-NMR (HSQC) analysis

**Figure 4:**
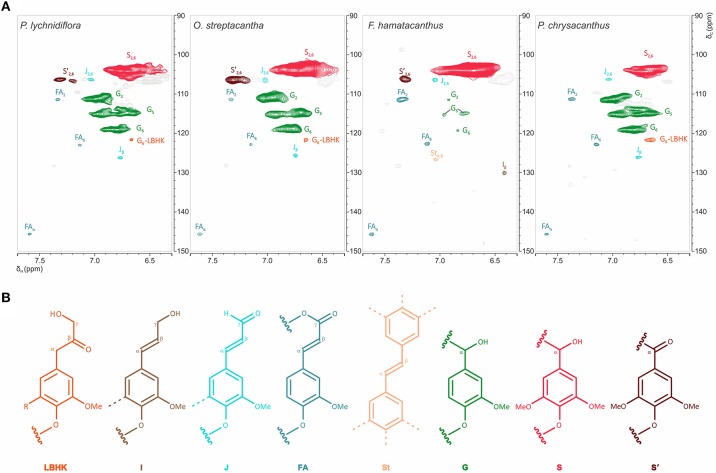
Structural features of the aromatic region of the wood lignin for the species of Cactaceae studied. **(A)** Aromatic/unsaturated region (δ_C_/δ_H_ 90–150/6.3–7.7 ppm) in the HSQC spectra. **(B)** Structures of the main aromatic units identified. In the LBHK structures: R = G, G-LBHK; R = S, S-LBHK (the corresponding signals were overlapped with others related to S units). Assignments for the cross-peaks in each structure are shown in Table 2.

The HSQC experiments allowed the estimation of relative abundances of the main types of inter-unit linkages in lignin, as well as those of the G and S units and other aromatic units. The HSQC spectra corresponding to the aliphatic oxygenated region (δ_C_/δ_H_ 50-90/2.7-5.6 ppm) as well as the representation of the identified structures, are presented in Figure 3. The HSQC spectra corresponding to the aromatic/unsaturated region (δ_C_/δ_H_ 90–150/6.3–7.7 ppm) as well as the representation of the identified structures are shown in Figure 4. Assignments for the cross-peaks found in both regions of the HSQC spectra are listed in Table 2. Relative abundances of the end-groups and the main inter-unit linkages, as well as the γ-acylation percentage, molar abundances of ferulates, stilbenes, G and S units of lignin and S/G ratio are given in Table 3.

**Table 2:** Assignments for the ^13^C–^1^H cross-peaks found in the HSQC spectra of DL from the wood in the species of Cactaceae studied.

**Label**	**δ_C_/δ_H_**	**Assignment**
B_β_	53.1/3.46	C_β_-H_β_ in phenylcoumaran structures (B)
C_β_	53.8/3.07	C_β_-H_β_ in β-β resinol structures (C)
OMe	55.9/3.74	C–H in methoxyls
F_β_	58.0/3.08	C_β_-H_β_ in spirodienone structures (F)
A_γ_	59.7/3.40 and 3.70	C_γ_-H_γ_ in β-O−4′ structures (A)
I_γ_	61.3/4.14	C_γ_-H_γ_ in in cinnamyl alcohol end-groups (I)
B_γ_	62.8/3.70	C_γ_-H_γ_ in phenylcoumaran structures (B)
${\text{A}}_{\text{γ}}^{\prime }$	63.5/4.22	C_γ_-H_γ_ in γ-acylated β-0-4′ structures (A′)
LBHK_γ_	67.2/4.16	C_γ_-H_γ_ in lignin-bound Hibbert ketone structures (LBHK)
C_γ_	71.1/3.82 and 4.19	C_γ_-H_γ_ in β-β′ resinol structures (C)
${\text{C}}_{\text{γ}}^{\prime }$	71.2/4.23 and 71.3/3.90	C_γ_-H_γ_ in γ-acetylated guaiacyl-syryingaresinol complexes (C′)
A_α_ (G)	71.7/4.67	C_α_-H_α_ in β-0–4′ structures (A) linked to G
A_α_ (S)	72.1/4.87	C_α_-H_α_ in B–O−4′ structures (A) linked to S
${\text{F}}_{{\text{β}}^{\prime }}$	77.2/4.62	${\text{C}}_{{\text{β}}^{\prime }}$-${\text{H}}_{{\text{β}}^{\prime }}$ in spirodienone structures (F)
E_α_	79.5/5.49	C_α_-H_α_ in α-O−4′ structures (E)
${\text{A}}_{\text{β}}^{\prime }$ (G)	80.8/4.51	C_β_-H_β_ in γ-acylated β-O−4′ structures (A′) linked to G
D_α_	83.3/4.82	C_α_-H_α_ in dibenzodioxocin structures (D)
Aox_β_	83.4/5.25	C_β_-H_β_ in α-oxidized β-O−4′ structures (Aox)
${\text{F}}_{{\text{α}}^{\prime }}$	83.5/4.72	${\text{C}}_{{\text{α}}^{\prime }}$-${\text{H}}_{{\text{α}}^{\prime }}$ in spirodienone structures (F)
A_β_ (G)	83.8/4.30	C_β_-H_β_ in β-O−4′ structures (A) linked to G
F_α_	84.0/5.28	C_α_-H_α_ in spirodienone structures (F)
C_α_	85.2/4.66	C_α_-H_α_ in resinol structures (C)
D_β_	85.3/3.87	C_β_-H_β_ in dibenzodioxocin structures (D)
${\text{C}}_{\text{α}}^{\prime }$	85.3/4.75	C_α_-H_α_ in γ-acetylated guaiacyl-syryingaresinol complexes (C')
A_β_ (S)*-e*	86.1/4.12	C_β_-H_β_ in *erythro* β-O−4′ structures (A) linked to S
A_β_ (S)*-t*	86.9/4.02	C_β_-H_β_ in *threo* β-O−4′ structures (A) linked to S
B_α_	87.0/5.46	C_α_-H_α_ in phenylcoumaran structures (B)
S_2,6_	104.2/6.70	C_2,6_-H_2,6_ in etherified syringyl units (S)
J_2,6_ (S)	106.5/7.04	C_2_-H_2_ and C_6_-H_6_ in sinapaldehyde end-groups (J)
S′_2,6_	106.6/7.32 and 7.22	C_2,6_-H_2,6_ in α oxidized syringyl units (S')
G_2_	111.2/7.00	C_2_-H_2_ in guaiacyl units (G)
FA_2_	111.4/7.36	C_2_-H_2_ in ferulates (FA)
G_5_	114.9/6.73 and 115.3/6.95	C_5_-H_5_ in guaiacyl units (G)
G_6_	119.1/6.81	C_6_-H_6_ in guaiacyl units (G)
G_6_-LBHK	121.8/6.65	C_6_-H_6_ in lignin-bound Hibbert ketone structures derived from G (G-LBHK)
FA_6_	123.5/7.02	C_6_-H_6_ in ferulate (FA)
J_β_	126.1/6.81	C_β_-H_β_ in cinnamyl aldehyde end-groups (J)
St_α, β_	126.6/7.04	C_α_-H_α_ and C_β_-H_β_ in stilbene structures (St)
I_β_	130.1/6.41	C_β_-H_β_ in in cinnamyl alcohol end-groups (I)
FA_α_	145.4/7.50	C_α_-H_α_ in ferulate (FA)
J_α_	153.7/7.61	C_α_-H_α_ in cinnamyl aldehyde end-groups (J)

*The HSQC spectra are shown in Figures 3, 4*.

**Table 3:** Relative abundances of the structures found in the HSQC spectra of DL for the species of Cactaceae studied, corresponding to the main inter-unit linkages and the main aromatic units of lignin.

	**Pereskioideae**	**Opuntioideae**	**Cactoideae**	
	***P. lychnidiflora***	***O. streptacantha***	***F. hamatacanthus***	***P. chrysacanthus***
**LIGNIN INTER-UNIT LINKAGES (%)**				
β-O-4′ (A)	80	85	67	70
α-Oxidized β-O-4′ (Aox)	1	3	0	0
Phenylcoumaran (B)	6	1	0	7
Resinol (C)^a^	13	10	26 (8)	22
Dibenzodioxocin (D)	0	0	1	1
α-O-4′ (E)	0	0	1	0
Spirodienone (F)	0	0	5	0
**LIGNIN SIDE-CHAIN** γ**-ACYLATION (%)**				
γ-Acylated β-O-4′ (A')	0	0	4	5
**LIGNIN END-GROUPS (%)**^b^				
Cinnamyl alcohol end-groups (I)	0	0	2	0
Cinnamaldehyde end-groups (J)	2	4	2	2
**LIGNIN AROMATIC UNITS (%)**^c^				
G	38 (27)	31 (23)	6 (3)	54 (44)
S	62 (73)	69 (77)	94 (97)	46 (56)
S/G Ratio	1.6 (2.7)	2.2 (3.4)	16.4 (35.0)	0.9 (1.3)
Ferulate (FA)	2	3	8	1
Stilbenes (St)	0	0	1	0

a *Percentages of γ-acetylated guaiacyl-syringaresinol complexes (C') are shown in parentheses*.

b *Expressed as a fraction of the total lignin inter-unit linkage types*.

c *Expressed as the number of units per 100 aromatic units; rounded percentages. The raw percentages, obtained earlier with nitrobencene oxidations, are shown in parentheses (obtained from the Supplementary Data in Reyes-Rivera et al., 2015)*.

#### Side-chain region

The most abundant inter-unit linkages were of the β-O−4′ ether-type (structure A, Figure 3), which range from 67% in *F. hamatacanthus* to 85% in *O. streptacantha* (Table 3). Cross-peaks at δ_C_/δ_H_ 63.5/4.22 and 80.8/4.51, corresponding to the C_γ_-H_γ_ and C_β_-H_β_ pairs, respectively, in the γ-acylated β-O−4′ ether structures (A′) were observed in *F. hamatacanthus* and *P. chrysacanthus* (Figure 3 and Table 2). On the other hand, a certain degree of oxidation (1–3%) in the C_α_ of the β-O−4′ ether (Aox) structures was observed in *O. streptacantha* and *P. lychnidiflora*. The phenylcoumaran structures (B) were found in considerable percentages in *P. chrysacanthus* (7%) and *P. lychnidiflora* (6%), but were absent in *F. hamatacanthus*, where the S units are markedly predominant (S/G = 16.4). The resinol structures (C) were secondarily abundant (10–26%) and its relative abundance showed a significant negative statistical correlation with the content of the β-O−4′ ether structures (*P* < 0.01; *r* = −0.994). In addition, a pair of cross-peaks were observed at δ_C_/δ_H_ 71.2/4.23 and 71.3/3.90 ppm and another one at δ_C_/δ_H_ 85.3/4.75 ppm, which were tentatively assigned to the pair of C_γ_-H_γ_ and the C_α_-H_α_ cross-peaks, respectively in the γ-acetylated guaiacyl-syryingaresinol complexes (structure C′, Figure 3 and Table 2), based on Ralph S. A. et al. (2004).

Dibenzodioxocin structures (D) were found in low proportions (1%) in *F. hamatacanthus* and *P. chrysacanthus* (Table 3), while α,β-diaryl ether (E) and spirodienone (F) structures were only found in *F. hamatacanthus*; the latter ones have an important contribution (5%) to the inter-unit linkages of the lignin. None of these structures was observed in *O. streptacantha* and *P. lychnidiflora*. Cross-peaks observed at δ_C_/δ_H_ 67.67/4.17 ppm were assigned to the C_γ_-H_γ_ in the lignin-bound Hibbert ketone structures (LBHK, Figure 3), based on Miles-Barrett et al. (2016).

#### Unsaturated/aromatic region

The most prominent cross-peaks belong to the guaiacyl (G) units, etherified syringyl units (S) and oxidized syringyl units (S′) of the lignin (Figure 4 and Table 2). In addition, cross-peaks belonging to ferulates (FA), stilbenes (St), cinnamyl alcohol end-groups (I) and cinnamyl aldehyde end-groups (J) were found, as well as the cross-peaks assigned to lignin-bound Hibbert ketones structures derived from G units (G-LBHK, Figure 4 and Table 2). Based on the calculated relative abundances (Table 3), a predominance of the S units of lignin was observed in three species: *P. lychnidiflora* (62%), *O. streptacantha* (69%) and *F. hamatacanthus* (94%). On the other hand, *P. chrysacanthus* was the only species with a slight predominance of G units (54%). An interesting aspect is that the relative abundances of the G units and ferulates (FA) showed a significant negative statistical correlation (*P* < 0.05; *r* = −0.969). The cinnamyl aldehyde end-groups (J) and ferulates (FA) were detected in all the species, but the latter were relatively more abundant in the wood lignin of *F. hamatacanthus* (8%; Table 3). Likewise, cinnamyl alcohol end-groups (I) and stilbenes (St) were only observed in the wood lignin of *F. hamatacanthus*.

**Figure 5:**
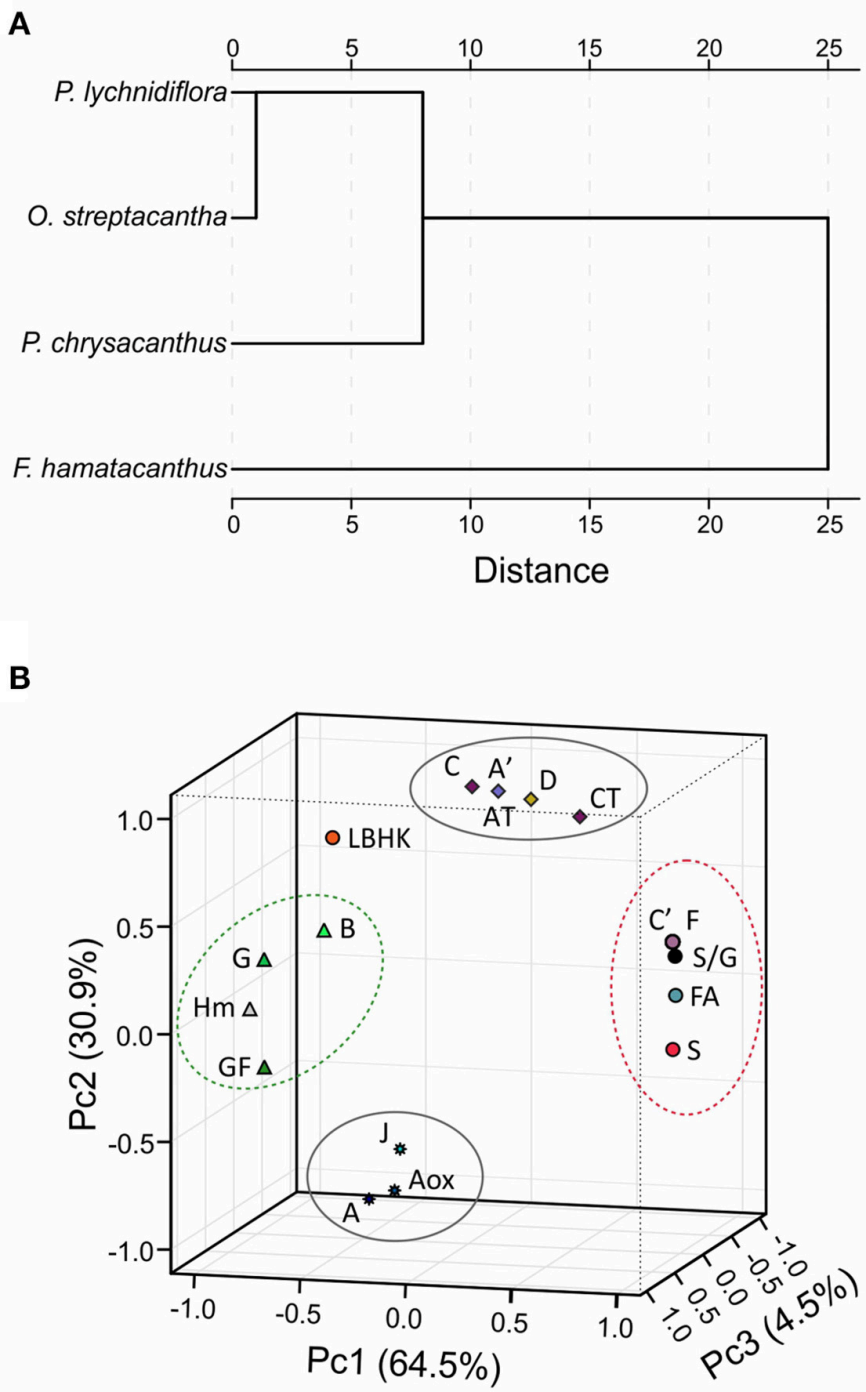
Results of chemometric analyzes using relative abundances from HSQC spectra. **(A)** Dissimilarity of the lignin of the four species, measured based on the square of the Euclidean distance. **(B)** Three-dimensional graphic obtained by the principal component analysis using matrices including discrete variables. Letters correspond to the structures in Figures 3, 4. AT, total abundance of β-O−4′ ether structures; CT, total abundance of resinol structures; GF, growth form; Hm, height of the species. Structures E, I and St overlapped with structures C′ and F.

Based on the Euclidean similarity, the hierarchical cluster analysis grouped the wood lignin of *O. streptacantha* and *P. lychnidiflora* together, while *P. chrysacanthus* and *F. hamatacanthus* were placed independently (Figure 5A). In the principal component analysis, the eigenvalues for three components were obtained for both matrices; those including discrete characters and those excluding them, and no significant differences were found (data not shown). The component 1 was the most informative, explaining around the 64.5% of the total variance. This was determined in both analysis by the features related with the wood anatomy (i.e., abundance of ferulates and resinol structures, including γ-acetylated guaiacyl-syringaresinol complexes) and those related with the growth form (i.e., species height, growth form and abundances of the G units and phenylcoumaran structures; Figure 5B).

### ATR-FTIR analysis

**Figure 6:**
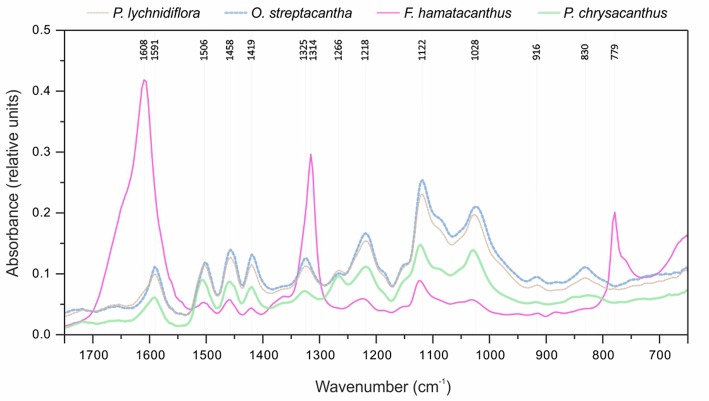
Raw ATR-FTIR spectra for the four species of Cactaceae studied.

**Figure 7:**
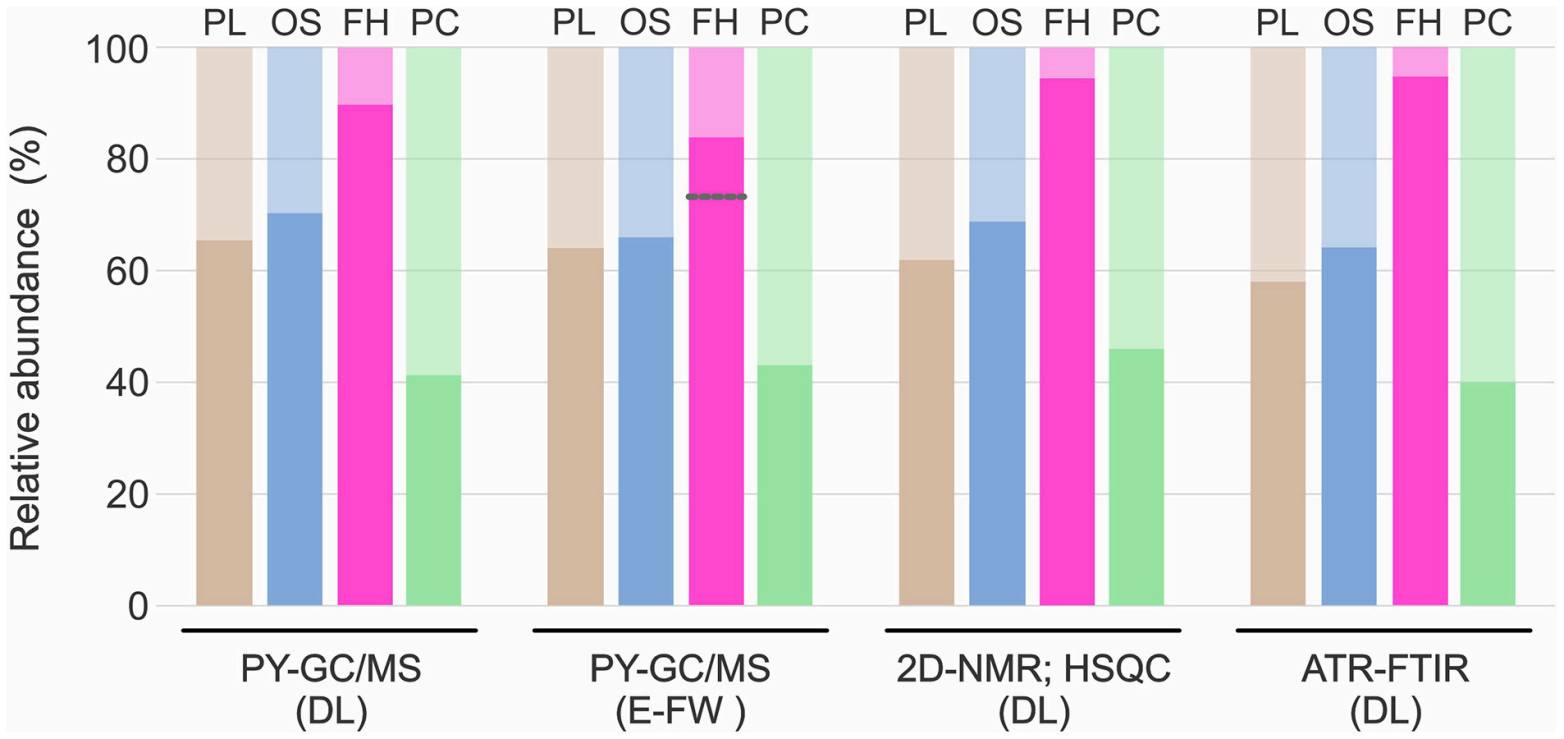
Relative abundances of G and S units obtained through the three methods. Standard deviation was always <10% of the average; except for the analysis of the E-FW by Py-GC/MS of *F. hamatacanthus*, where there was a higher deviation induced by the abundance of 4-vinylguaiacol. Darker color in each bar represents S units and lighter color G units. Gray line indicates the relative abundance when 4-vinylguaiacol was included. FH, *F. hamatacanthus*; OS, *O. streptacantha*; PC, *P. chrysacanthus*; PL, *P. lychnidiflora*.

Through the analysis of the DL by ATR-FTIR differences in the functional groups between species were evaluated and the S/G ratio was calculated. The raw spectra obtained by ATR-FTIR are shown in Figure 6. Peaks were detected at 1,608, 1,506, 1,458, and 1,122 cm^−1^, assigned to aromatic rings vibrations. Especially in *F. hamatacanthus* three very high peaks, associated with the S units, were observed (Figure 6). The peak at 1,608 cm^−1^ was assigned to C = C stretching in the aromatic rings of S units, the peak at 1,314 cm^−1^ was assigned to the vibration of the C1–O in the derivatives of S units and the peak at 779 cm^−1^ was assigned to the meta-di-substituted benzene groups. On the other hand, the peaks at 1,266 and 1,218 cm^−1^, assigned, respectively to the in-plane vibrations of the C–H and C–O–C in the G units, were more noticeable in the other species, mainly in *P. chrysacanthus*. A similar behavior was observed in the peaks at 1,028 cm^−1^, assigned to the stretching of the C–O–C ether linkages; in the peaks at 919 cm^−1^, assigned to the out-of-plane deformations in the aromatic C–H; and in the peaks at 830 cm^−1^, assigned to the out-of-plane vibrations in the C–H in the G units. Due to the overlapping of some peaks, the S/G ratio could not be calculated on the raw ATR-FTIR spectra; however, the correct estimation of the abundances of the G and S units was achieved through spectra deconvolution (Figure S2). The obtained S/G ratios were highly consistent with those obtained for the DL and E-FW by 2D-NMR (HSQC) and Py-GC/MS (Figure 7). The hierarchical cluster analysis using the absorbance of the peaks in the region between 750 and 1,750 cm^−1^ recovered the same groups than those obtained with the data from the HSQC experiments.

## Discussion

### Low γ-acylation in the wood lignin of cactaceae

Py-GC/MS is a coupled semi-quantitative technique that allows knowing the phenols resulting from cleavage of ether and certain carbon-carbon linkages of the lignin by the effect of thermal degradation (Evtuguin et al., 2001; del Río et al., 2004; Rencoret et al., 2011). Produced phenols retain their substitution patterns, thus relative abundances of the different lignin monomers can be successfully quantified (Rencoret et al., 2008, 2009, 2011, 2015; Lourenço et al., 2016). In addition, it allows for the recognition of γ-acylated monolignols, such as coniferyl- and sinapyl acetates (del Río et al., 2004), both related to drought-resistant plants (del Río et al., 2004; Ralph, 2005, 2010). As shown in the results of the Py-GC/MS of DL (Figure 2 and Table 1) and E-FW (Figure S1 and Table S1), coniferyl- and sinapyl acetates are not present in the lignin of the Cactaceae studied here. The limited acylation in the side-chains of lignin is given mainly by the γ-acetylated carbons in the G units, as observed in the HSQC spectra of *F. hamatacanthus* and *P. chrysacanthus* (C_β_-H_β_ cross-peaks at δ_C_/δ_H_ 80.8/4.51 ppm; structures A′ and C′, Figure 3). In addition, lignins with a high content of S units are low or no γ-acylated (Table 3). These two important traits contrast with that reported by other authors, which have related the extensive γ-acylation in lignin with a high content of S units (Ralph, 1996, 2010; del Río et al., 2004) and with a decreasing loss of water by improving of the hydrophobicity of the vascular tissue (Ralph, 2005, 2010; del Río et al., 2007). In the case of Cactaceae, the species studied here are, as the majority of this family, exposed to hydric stress and very long drought periods. Thus, the low or no γ-acylation of the wood lignin in Cactaceae is evidence that alternate adaptive routes have been followed in this family of succulent plants to solve negative effects of drought and excessive water loss. Those ways do not imply necessarily the recruitment of high proportions of γ-acylated monolignols in the wood lignin. Rather, they involve physiological and morpho-anatomical adaptations for improvement of the water conduction/retention, such as an efficient use of water (Edwards and Donoghue, 2006; Ogburn and Edwards, 2009), succulence in cortical and woody tissues (Mauseth, 2006), wood anatomy and the morphology of wood elements (Figure 1; Altesor et al., 1994; Mauseth and Plemons, 1995; Loza-Cornejo et al., 2003; Vázquez-Sánchez and Terrazas, 2011; Reyes-Rivera et al., 2015, 2017). Additionally, the low or no γ-acylation in the S-rich wood lignins in Cactaceae can be due to an enzymatic mechanism not preponderant in this group. It has been suggested that the high γ-acylation of lignin could be a mechanism to achieve S-rich lignins (Ralph, 1996; del Río et al., 2004). For example, the S-rich lignin of some monocots is highly γ-acylated (58–80%, del Río et al., 2007, 2008). Especially the S units of abaca (*Musa textilis*; 45% γ-acylated) and caraua (*Ananas lucidus*; 15% γ-acylated) are predominantly γ-acylated by *p-*coumarates (del Río et al., 2007). A key aspect is that both abacá and caraua are non woody species belonging to relatively close groups (clade Commelinidae). In contrast, the limited γ-acylation of G units is dispersed along the plants, the same as the extensive γ-acylation with acetate groups in S units (del Río et al., 2007). Therefore, it can be regarded that the enzymatic mechanism responsible of the extensive γ-acylation of lignin (i.e., formation of previously γ-acylated cinnamyl alcohols and their subsequent incorporation into lignin, Lu and Ralph, 2008; or the incorporation of p-coumarates as pending-terminal groups, Ralph, 2010) is genetically fixed only in some plant groups and that, at least in Cactaceae, it is not a strictly necessary way for the formation of S-rich lignins.

### Lignin structure as revealed by 2D-NMR (HSQC)

2D-NMR is a powerful tool for the characterization of the lignin structure (Capanema et al., 2005; Mansfield et al., 2012; Constant et al., 2016), it has been successfully used to calculate the relative abundances of the inter-unit linkages in the side-chains of the lignin, including benzodioxane, dibenzodioxocins and spirodienones structures, and those of the H, G, S, and catechyl aromatic units, among other monolignols (Zhang et al., 2006; Rencoret et al., 2009; Ralph, 2010; Chen et al., 2013; Carlos Del Río et al., 2017).

#### Main inter-unit linkages in the side-chain region

The abundance patterns of the main inter-unit linkages in the wood lignin of the studied Cactaceae are similar to those reported for other angiosperms (Table 3). However, an interesting aspect is the relationship observed between the S/G ratio and the relative abundances of phenylcoumaran (B) and resinol (C) structures. It has been suggested that the nature of the monolignols influences on both lignin composition and inter-unit linkages abundances (Rencoret et al., 2011; Meents et al., 2018); e.g., sinapyl alcohol favors the formation of β-β′ linkages of the resinol structures (Ralph J. et al., 2004). The results shown here contrast partially with that fact: in species with contrasting abundances of S units (*F. hamatacanthus*, 94% and *P. chrysacanthus*, 46%) very similar abundances of resinol structures (26 and 22%, respectively) were obtained. A possible explanation for this fact is that β-β′ linkages act as starting points for the growth of the lignin polymer, so they would be formed only during a determined period in the lignification (Ralph J. et al., 2004). An aspect that seems to support this hypothesis is the relationship between γ-acetylated guaiacyl-syringaresinol complexes and ferulates acting as nucleation sites for lignification, which is detailed below.

Other structures with low proportions were found in the wood lignin of the Cactaceae studied, such as α-oxidized β-O−4′ ether (Aox), counting around 3% of side-chain linkages (Figure 3 and Table 3). Although the functional role of these structures in lignin has not been concretely demonstrated, in tracking studies comparing wood lignin in young vs. adult individuals of *Eucalyptus globulus*, α-oxidized β-O−4′ ether structures were related to the wood aging (Rencoret et al., 2011). A specially intriguing aspect is that in this study α-oxidized β-O−4′ ether structures were only found in the wood lignin of *O. streptacantha* and *P. lychnidiflora*, both having a low degree of succulence in the stem (Figure 1). In contrast, these structures were not found in the species of Cactoideae, *F. hamatacanthus* and *P. chrysacanthus*; both species have a greater degree of succulence in the stem (Figure 1). Thus, the absence of these structures could be indirectly related to metabolic processes against oxidative damage in species with considerably succulent stems.

#### Branching in side-chains of the S-rich lignin

Spirodienone structures (F) have been identified previously in isolated lignin from different gymnosperms and angiosperms (Zhang et al., 2006; Rencoret et al., 2008; del Río et al., 2012; Wen et al., 2013; Lourenço et al., 2016). Two forms of spirodienone structures have been described, those formed by G units, and their counterparts, more resistant to degradation, formed by S units (Zhang et al., 2006); both are considered as branching-linkages in the side-chains of lignin (Mar and Kulik, 2017). In this study, spirodienone structures were only found in the wood lignin of *F. hamatacanthus*, they were formed by S units (Figure 3 and Table 3) and have an important contribution in side-chain linkages (5% of abundance). A key aspect is that lignin of this species has a high proportion of S units, which makes its structure very linear; this would imply that the function of the spirodienone structures in the S-rich lignin of *F. hamatacanthus* wood is also to contribute as a branching-linkage.

#### The role of the S-rich lignin within cactaceae

In a previous exploratory study, the S/G ratios determined by nitrobenzene oxidations for thirteen species of Cactaceae were published (Reyes-Rivera et al., 2015). An intriguing aspect of that work was the predominance of S-rich lignins all over the Cactaceae species; i.e., raw percentages of S units ranged from 50 to 97% (supplementary data published by Reyes-Rivera et al., 2015). In this work, the 2D-NMR analysis of the DL allowed the estimation of more realistic relative abundances of G and S units (Table 3). Based on the results for the selected species in this work and the previously published results (Reyes-Rivera et al., 2015), it can be regarded that S-rich lignins predominate in Cactaceae woods, especially in dimorphic woods (Table 3; Reyes-Rivera et al., 2015). Recent studies have attributed a protective function to S-rich lignins in both monocots (Menden et al., 2007) and dicots (Skyba et al., 2013). In wheat (*Triticum aestivum*) cultivars that are highly resistant to infections by the rust fungi (*Puccinia graminis* f. sp. *tritici)* it was observed that the attack is stopped by the hypersensitive response accompanied by the intercellular accumulation of S-rich lignin (Menden et al., 2007). Likewise, in *Populus alba* × *tremula* it was shown that the highly linear structure of the S-rich lignin results in a more compact arrangement, forming ligno-celullosic complexes more recalcitrant to the attack by oxidative agents such as wood decay fungi (Skyba et al., 2013). Due to the high succulence surrounding the wood of *F. hamatacanthus* and most of the species of Cactaceae, it is highly probable that S-rich lignins are a defense strategy against pathogens or against oxidative stress from lignification itself.

#### Lignification and decreasing of oxidative damage in cactaceae wood

The oxidative damage suffered by cells during lignification, at the cytoplasm or cell wall level, is prevented by some NADPH-dependent reductases through the reduction of products derived from the oxidative coupling of monolignols (Niculaes et al., 2014; Nuoendagula et al., 2016; Meents et al., 2018). A fact that supports the role of S-rich lignins in the decreasing of oxidative damage in Cactaceae is the finding of a considerable number of candidate genes coding NADPH-dependent reductases (i.e., Phenylcoumaran Benzylic Ether Reductase, PCBER, or Pinoresinol-Lariciresinol Reductase, PLR) in the cambial zone of several species, mainly in those with dimorphic wood (Figure S3; Table S2). PCBER prevent the formation of α-O−4′ linkages in the phenylcoumaran structures by reduction reactions (Niculaes et al., 2014). Thus, the activity of these enzymes, or other reductases, could be the reason for the different abundances of phenylcoumaran structures in the species with similar abundances of S units such as *O. streptacantha* and *P. lychnidiflora* (Table 3). The high metabolic plasticity observed in lignification enables the influence of different factors from secondary metabolism on the final structure of lignin (Lu and Ralph, 2002; Bonawitz and Chapple, 2010; Ralph, 2010; Carlos Del Río et al., 2017). Thus, the formation of mono-, di- or oligolignols and their later incorporation into lignin polymer would be product of the active transcriptome in the protoplast of lignifying cell or neighboring parenchymatic cells in a cooperative lignification mechanism (Hosokawa et al., 2001; Pesquet et al., 2013; Meents et al., 2018).

#### Ferulates and the formation of lignin-carbohydrate complexes in dimorphic wood

**Figure 8:**
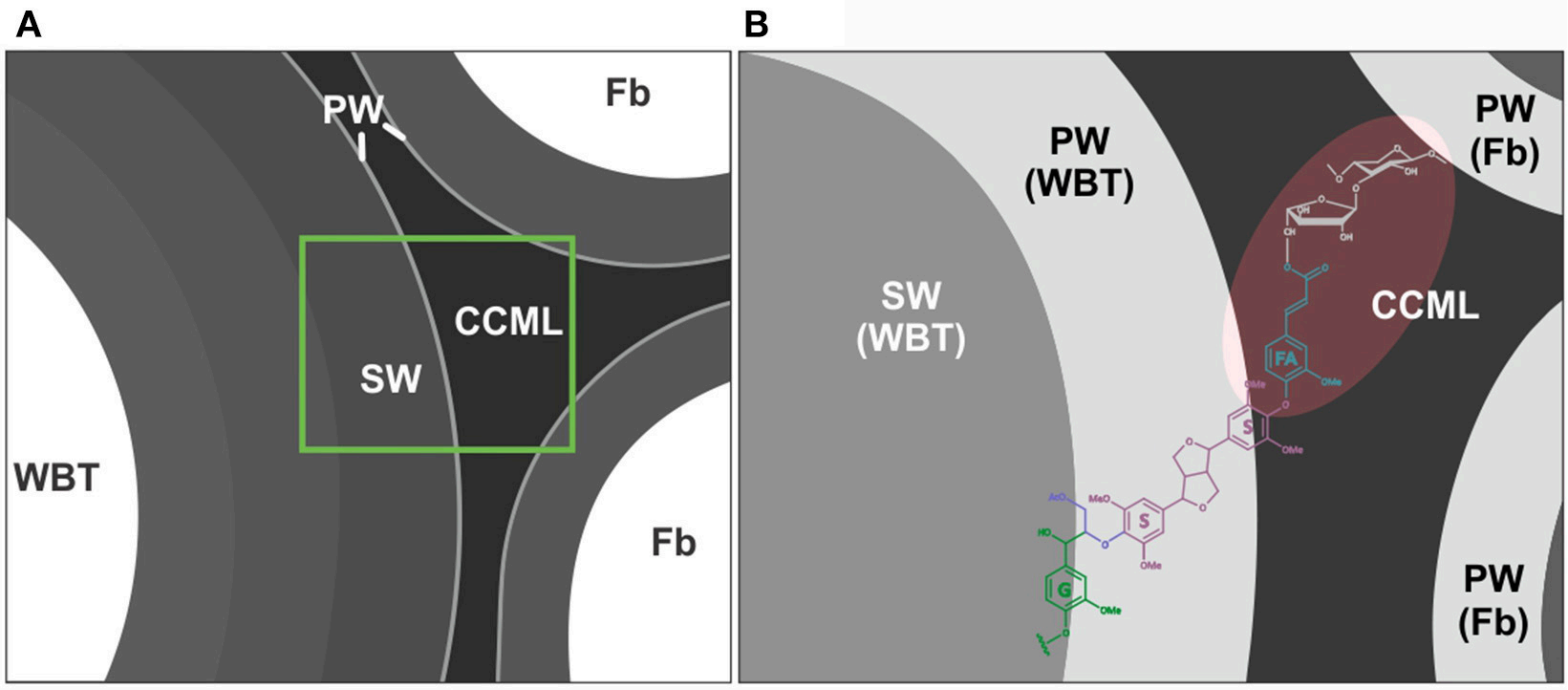
Role of the ferulates and γ-acetylated guaiacyl-syringaresinol complexes in the WBT-fiber junctions. **(A)** General representation of WBT-fiber junctions. **(B)** Ferulates would act as cross-links between carbohydrates and lignin. The nucleation sites of lignification (shaded in red) would include the corners of the compound middle lamella, between the WBT-fiber junctions. CCML, corner of the compound middle lamella; FA, ferulates; Fb, fibers; G, guaiacyl units (γ-acetylated); PW, primary cell wall; S, syringyl units; SW, secondary cell wall. A portion of the carbohydrate matrix is depicted in gray.

Ferulates are incorporated to the lignin via radical coupling reactions with mono- or oligolignols (Bunzel et al., 2004; Ralph, 2010). It has been suggested that ferulates act as cross-links between lignin (ether linkages) and carbohydrates (ester linkages) in the primary and secondary cell wall from different tissues and plant lineages (Sun et al., 1997; Ralph, 2010; Ralph and Landucci, 2010; del Río et al., 2012; Lourenço et al., 2016). Furthermore, they are considered as initiation/nucleation sites in the lignification, providing mechanical resistance and stability to the cell (Ralph, 2010; Ralph and Landucci, 2010). In this work it was observed that fibrous Cactaceae species showed relatively lower abundances of ferulates (≤3%, Table 3), whereas in *F. hamatacanthus* (a dimorphic species) considerable percentages (8%) of these units were found. Based on its relative abundances in the species studied here, it can be considered that ferulates play an important role in the cell-cell interactions within dimorphic wood of *F. hamathacanthus*, especially in the WBT-fiber junctions, as it had been proposed previously for dimorphic woods (Reyes-Rivera et al., 2015). An aspect that support the role of ferulates as initiation points in wood lignification of this species is that ferulates and γ-acetylated guaiacyl-syringaresinol complexes have the same relative abundances. Like ferulates, resinol structures formed by β-β′ linkages have been considered as starting points in lignification (Ralph J. et al., 2004). Altogether, this is evidence of the synchronous formation of both structures to act as nucleation/starting points in the lignification to confer mechanical strength and stability to the wood, mainly at the corners of the compound middle lamella between the WBT-fiber junctions, as exemplified in Figure 8.

#### Relative abundance of G and S units by different methods

In general, the relative abundances of the G and S units obtained in this work through the three different methods were highly consistent for each species (Figure 7). However, relative abundances of G units obtained by Py-GC/MS for the E-FW from *F. hamatacanthus* were highly overestimated, mainly because of the relatively high abundance of 4-vinylguaiacol (Compound 18, Figure S1 and Table S1), which can be derived from ferulates (del Río et al., 2012; Rencoret et al., 2015). On the other hand, relative abundances of G and S units obtained here are similar to those obtained previously by nitrobenzene oxydations (Reyes-Rivera et al., 2015), except for *P. chrysacanthus*, where it was found that a considerable portion of G units had been underestimated due to the high proclivity of G units to form condensed structures (Sarkanen and Hergert, 1971; Lapierre, 2010). Based on the relative abundances obtained, it is evident that high proportions of S units form the wood lignin in many Cactaceae, mainly in species with dimorphic wood (Figure 7, Reyes-Rivera et al., 2015). In a previous work tracking the lignification by the incorporation of isotope C^14^O_2_, it was shown that S units are formed more slowly than G units (Brown et al., 1953). On the other hand, the great abundance of G units in the lignin has been related to a rapid deposition (Lourenço et al., 2016). In an evolutionary context, the extraordinary high abundance of S units (higher than 90%) in the lignin from dimorphic wood in Cactaceae could be accentuated by the combination of two processes concerning to the wood lignification: structural defense/support mechanisms and the retardation of the developmental rates of the species (allometric neoteny *sensu*; Altesor et al., 1994).

## Conclusions

The information obtained by 2D-NMR, Py-GC/MS and ATR-FTIR has allowed to know more specific aspects of the lignification in the Cactaceae family, one of the succulent plant groups more spectacularly radiated within angiosperms. In this sense, the predominance of S-rich lignins in Cactaceae wood is confirmed; in species of xeric habitats (especially in small species with dimorphic wood), extreme conditions influence the cell wall structure, inducing a lignification typical of stressed plants with a hypersensitive response. In addition, evidence of a high association between S-rich lignin and the development of dimorphic wood is presented here: the γ-acetylated guaiacyl-syringaresinol complexes acting as nucleation sites of lignification in WBT-fiber junctions together with ferulates acting as cross-links between lignin and carbohydrates. On the other hand, the influence of other metabolic factors, such as reductases enzymes, on the nature of the derivatives of the phenylpropanoids and on the inter-unit linkages from the wood lignin is considered. Finally, the abundance of S units, the S/G ratio, resinol structures, spirodienones and ferulates are considered as highly informative for the study of adaptive-evolutionary processes in Cactaceae wood. Likewise, the highly informative capability of Py-GC/MS and ATR-FTIR for the estimation of relative abundances of guaiacyl and syringyl units in isolated lignin is highlighted.

## Author contributions

JR-R and TT designed the work. JR-R performed the experiments and prepared the figures. JR-R, TT, MS-H, and GC-E analyzed the data. TT, MS-H, GC-E, and JR-R provided reagents, materials and software. JR-R and TT wrote the manuscript. All the authors have read and approved the manuscript.

### Conflict of interest statement

The authors declare that the research was conducted in the absence of any commercial or financial relationships that could be construed as a potential conflict of interest.
