# Supplementary material for: Structural Characterization of Lignin in Four Cacti Wood: Implications of Lignification in the Growth Form and Succulence
Source: Front Plant Sci. 2018 Oct 17;9:1518. doi: 10.3389/fpls.2018.01518 (PMC6199501; doi:10.3389/fpls.2018.01518)
Supplement: Supplementary file 1 [file Data_Sheet_1.PDF]

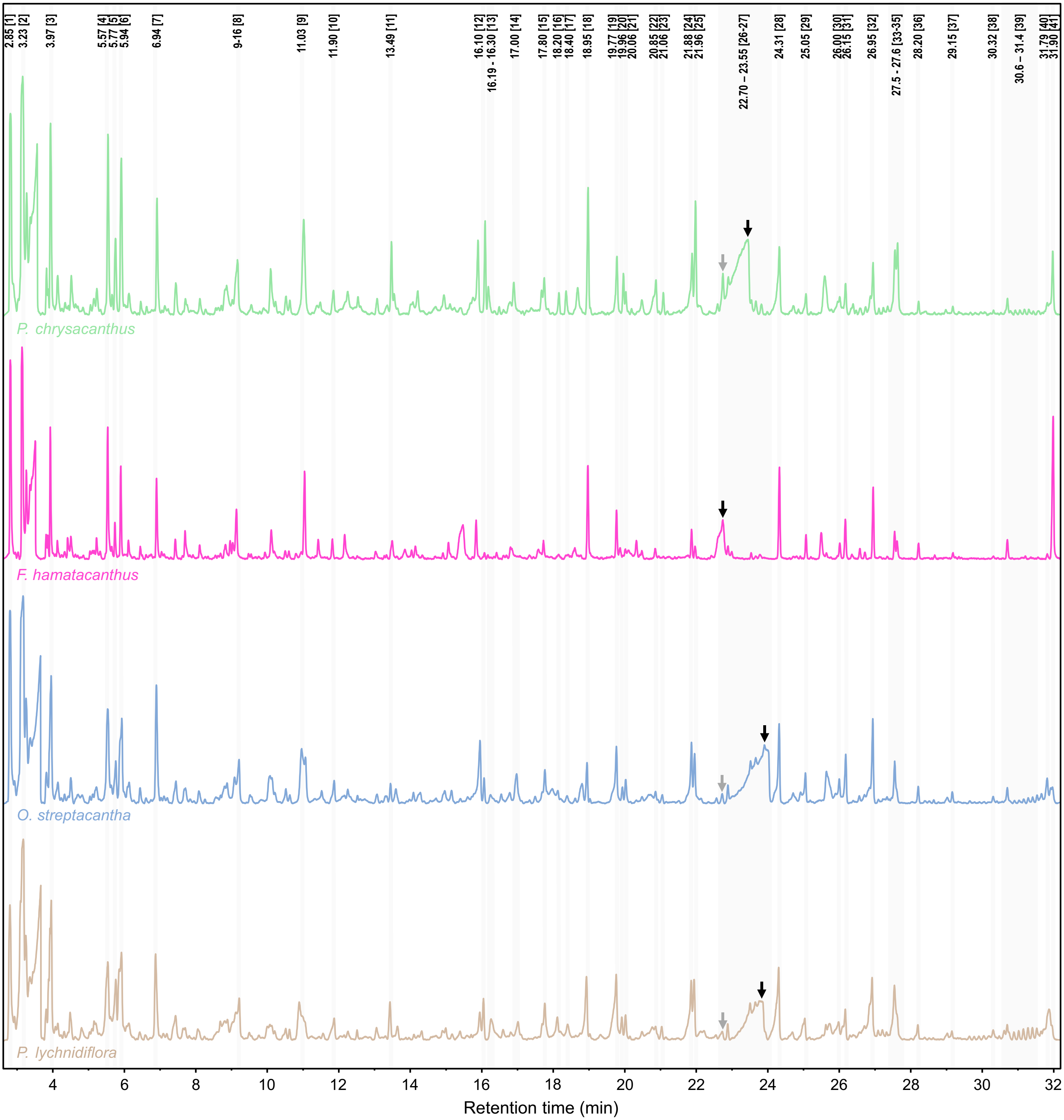

Figure S1. Characterization of the E-FW by Py-GC/MS for the species of Cactaceae studied**.** Identities and relative abundances of the released compounds are listed in **Table S1**. Gray arrows, acetovanillone; black arrows, levoglucosan.
