# Supplementary material for: Structural Characterization of Lignin in Four Cacti Wood: Implications of Lignification in the Growth Form and Succulence
Source: Front Plant Sci. 2018 Oct 17;9:1518. doi: 10.3389/fpls.2018.01518 (PMC6199501; doi:10.3389/fpls.2018.01518)
Supplement: Supplementary file 2 [file Data_Sheet_2.PDF]

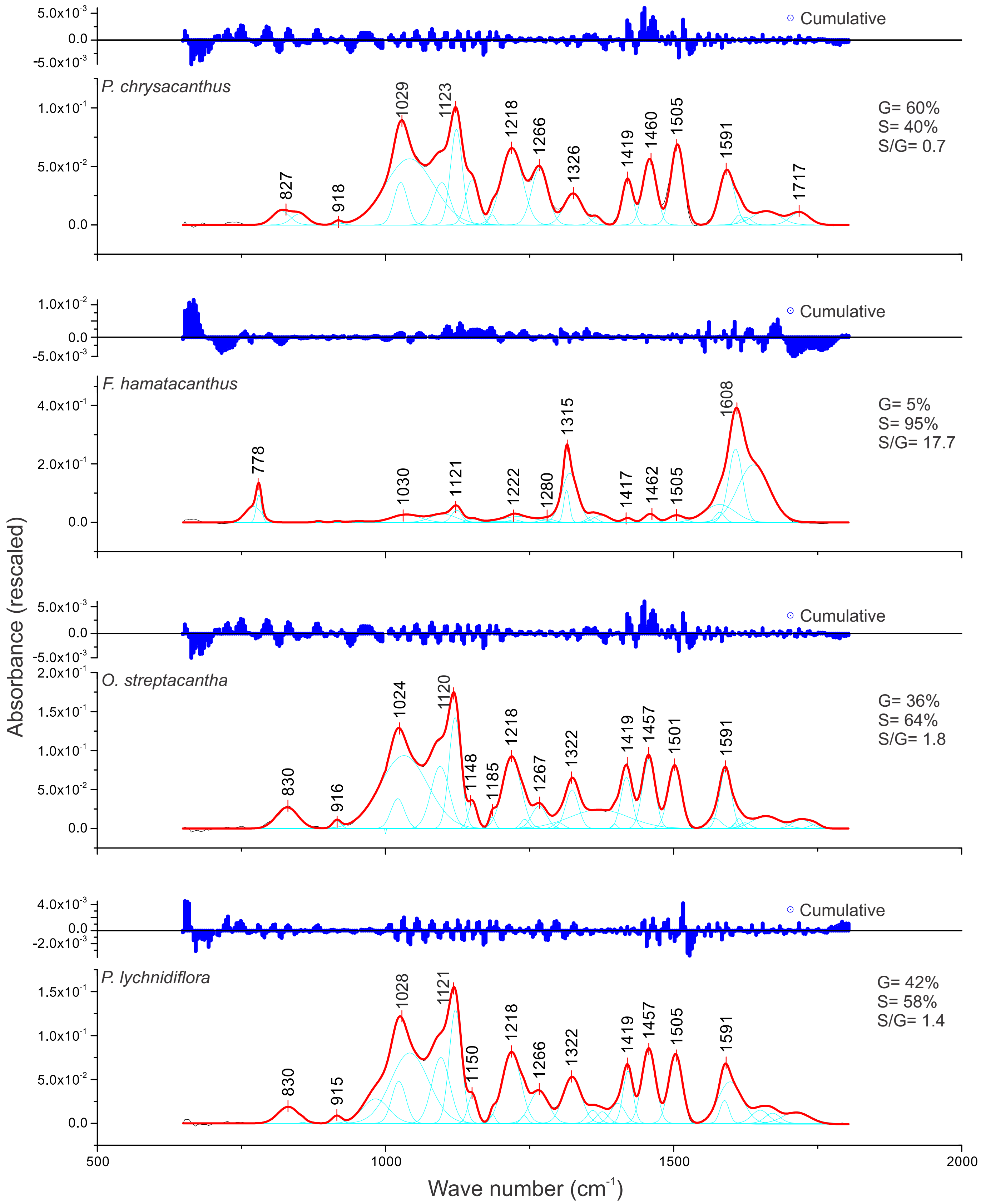

Figure S2. ATR-FTIR spectra deconvolution used to calculate relative abundances of G and S units, as well as the S/G ratio. The sum of the squares of the residual error for each spectrum was always in the range of $\pm 5.0x10^{-3}$, the determination coefficients were never less than 0.98.
