# Supplementary material for: Structural Characterization of Lignin in Four Cacti Wood: Implications of Lignification in the Growth Form and Succulence
Source: Front Plant Sci. 2018 Oct 17;9:1518. doi: 10.3389/fpls.2018.01518 (PMC6199501; doi:10.3389/fpls.2018.01518)
Supplement: Supplementary file 3 [file Data_Sheet_3.PDF]

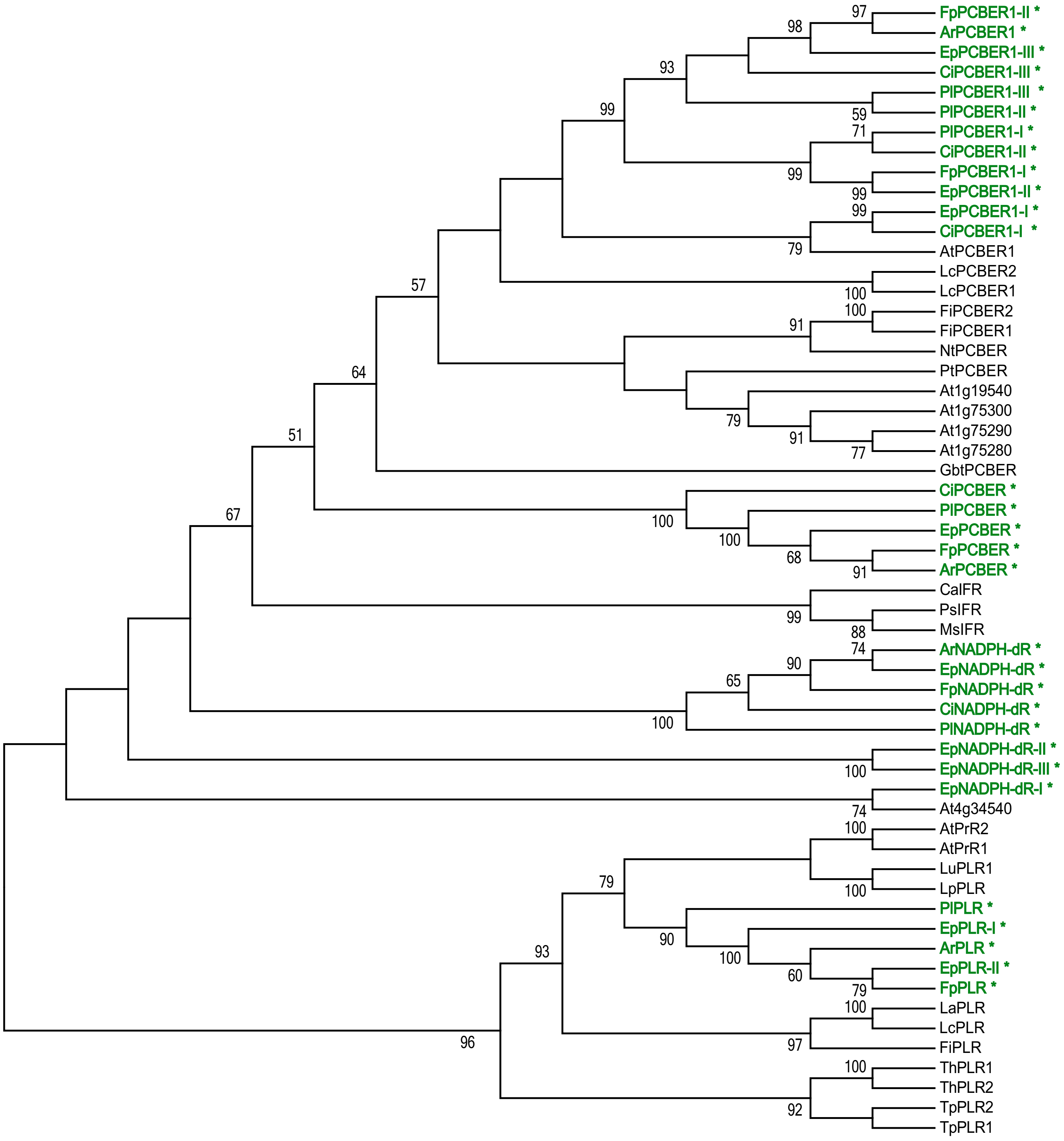

Figure S3. Phylogeny of the NADPH-dependent reductases derived from diverse plant species, including several Cactaceae species (green/asterisks); obtained by maximum likelihood following the method described in Reyes-Rivera et al. (2017). Abbreviations: PCBER, phenylcoumaran benzylic ether reductase; PLR, pinoresinol–lariciresinol reductase; IFR, isoflavone reductase. The data of each aminoacid sequence are shown in Table S2.
